# Impact of 16S rRNA on Intestinal Flora Alterations and Early Diagnosis in Early Alzheimer’s Disease Patients

**DOI:** 10.62641/aep.v53i2.1682

**Published:** 2025-03-05

**Authors:** Danping Lv, Xiuqin Lin, Xinyuan Zhang, Qundi Shen

**Affiliations:** ^1^Department of Laboratory, Shaoxing Seventh People’s Hospital, 312000 Shaoxing, Zhejiang, China

**Keywords:** Alzheimer’s Disease, 16S rRNA, intestinal microbiota, Early Alzheimer’s Disease, diagnosis

## Abstract

**Background:**

Alzheimer’s Disease (AD), a complex clinical condition, relies on neuropsychological assessments for early diagnosis. Recently, the gut-brain axis has been recognized as crucial in AD development, with dysbiosis in gut microbiota implicated in disease progression. Utilizing 16S rRNA analysis provides comprehensive monitoring of gut microbiota, potentially revealing biological markers for Early Alzheimer’s Disease (EAD). Therefore, this study aimed to investigate the diagnostic impact of 16S ribosomal RNA (rRNA) on changes in intestinal flora among EAD patients.

**Methods:**

This study analyzed stool samples from 50 AD patients and 50 healthy controls between June 2022 and June 2023. Based on the disease stage, patients were categorized into EAD (*n* = 14) and Late Alzheimer’s Disease (LAD) groups (*n* = 36). The V3–V4 region was sequenced using 16S rRNA quantitative Polymerase Chain Reaction (qPCR) to compare the composition of gut microbiota and differences in abundance among the three experimental groups.

**Results:**

The abundance and diversity of gut microbiota significantly increased in EAD patients compared to the healthy control group. Furthermore, 39 genera showed considerable variations between EAD and LAD patients and healthy controls, with notable increases in the abundance of *Bryantella*, *Gemmiger*, *Desulfovibrio*, *Collinsella*, and *Odoribacter* among EAD patients. Additionally, significant differences were observed across the *Desulfovibrioales *and *Verrucomicrobiales*, which could help distinguish EAD patients (Area Under the Curve (AUC) range 0.854, 0.966, *p* < 0.05).

**Conclusion:**

16S rRNA technology can be used to identify EAD patients, with the *Desulfovibrioales* and* Verrucomicrobiales* indicators serving as potential biological markers.

## Introduction

Alzheimer’s Disease (AD) significantly affects the physical and psychological health of the elderly [1, 2]. According to the International Alzheimer’s Association, the total cost of healthcare, long-term care, and end-of-life services for AD patients over 65 was estimated at $321 billion in 2022 [3]. The “China Alzheimer’s Disease Report” [4] indicated that in 2019, the prevalence of AD and other dementias was 924.1 per 100,000, with a mortality rate of 22.5 per 100,000.

AD is a pathologically complex, progressive, and fatal disease [5]. The mechanisms underlying AD are not fully understood, and early diagnosis remains challenging. Subjective assessment methods, such as the Montreal Cognitive Assessment and Brain Health Survey, have been primarily used for early diagnosis, making early detection and intervention difficult [6]. However, subjective diagnosis methods are susceptible to interference from multiple factors, resulting in insufficient diagnostic accuracy [7]. Therefore, identifying a non-invasive diagnostic protocol for Early Alzheimer’s Disease (EAD) is critical for the prevention and treatment of AD.

It is widely accepted that patients experience a phase of silence and early symptoms before irreversible cognitive decline, leading to debilitating disease [8]. Early identification of mild cognitive dysfunction is a crucial step in preventing the occurrence and progression of AD [9]. Recent research has reported intestinal barrier dysfunction in AD patients, which can be related to its etiology [10]. The microbiota, proteomic, and molecular changes in the gut occur long before symptoms appear. Moreover, differences between the gut microbiota of normal-aging individuals and mice with advanced AD highlight the potential of these critical hyphae, proteins, and pathways as markers for both the early and late stages of AD [11]. It has been suggested that gut microbial composition can be used not only to diagnose neurodegenerative and neurodevelopmental diseases early but also to alter the gut microbiota to influence the microbiota-gut-brain axis, providing a therapeutic target for refractory diseases [12]. Neuroinflammation induced by intestinal dysbiosis and lipid metabolism disorders plays a significant role in the progression of AD [13]. Furthermore, the gut is a major source of bacteria and antigens responsible for neuroinflammation following brain injury [14]. Therefore, considering the intestinal flora of AD patients as a monitoring index for screening and diagnosis of EAD is useful.

16S ribosomal RNA (rRNA) gene sequencing, a fundamental approach in microbial community profiling, leverages next-generation sequencing (NGS) platforms to elucidate the taxonomic composition and phylogenetic relationships of complex microbial populations within fecal samples [15, 16]. This approach has been extensively applied to patients with diabetes mellitus who also exhibit neurological dysfunction [17], epilepsy [18], and cognitive disorders [19] to determine the degree of intestinal flora disturbance and the specific location of bacterial distribution [20]. Animal experiments have revealed substantial changes in the abundance of intestinal flora in AD mouse models, including a decrease in Proteus, Enterococcus, Dulibacterium, and Ruminococcus, and an increase in pseudomonas [21]. Furthermore, research has revealed that degenerative cervical spondylosis causes dynamic changes in the intestinal flora over time, reducing the number of butyl-ureas and lactic acid-producing bacteria [22]. However, there is a considerable paucity of research on the use of 16S rRNA assays to identify neuropathological areas in Early Alzheimer’s Disease (EAD). Therefore, employing high-throughput quantitative detection technologies that focus on 16S rRNA is crucial for fully understanding the role of intestinal flora in the early diagnosis of Alzheimer’s Disease (AD). This study utilized quantitative Polymerase Chain Reaction (qPCR) based on 16S rRNA to extensively monitor changes in the intestinal microecology of 50 AD patients and control groups, aiming to assess its effectiveness for EAD detection. The existing literature also indicates a scarcity of data on applying 16S rRNA gene sequencing in pinpointing EAD neuropathological regions. Consequently, adopting high-throughput quantitative detection technologies based on 16S rRNA analysis is essential for elucidating the prognostic significance of gut microbiota in the early detection of EAD. Additionally, this study strategically utilized qPCR targeting 16S rRNA to carefully track and document fluctuations in the intestinal microbiota of the study cohort. This study aimed to determine the potential utility of 16S rRNA in the context of EAD. Furthermore, this study aims to discover new biomarkers and improve the early identification of AD, providing a biological basis for early intervention and treatment.

## Materials and Methods

### Recruitment of Study Subjects

This study included stool specimens of 50 AD patients who visited the Shaoxing Seventh People’s Hospital, China, from June 2022 to June 2023, and 50 healthy individuals who visited during the same period were selected as the control group. The ratio of age and gender between AD patients and healthy controls was 1:1 utilizing the propensity score method. The study design was approved by the Medical Ethics Committee of the Shaoxing Seventh People’s Hospital, China and was performed following the Declaration of Helsinki (ethical approval number: 2022-013). All study subjects signed the informed consent form.

**Fig. 1.**
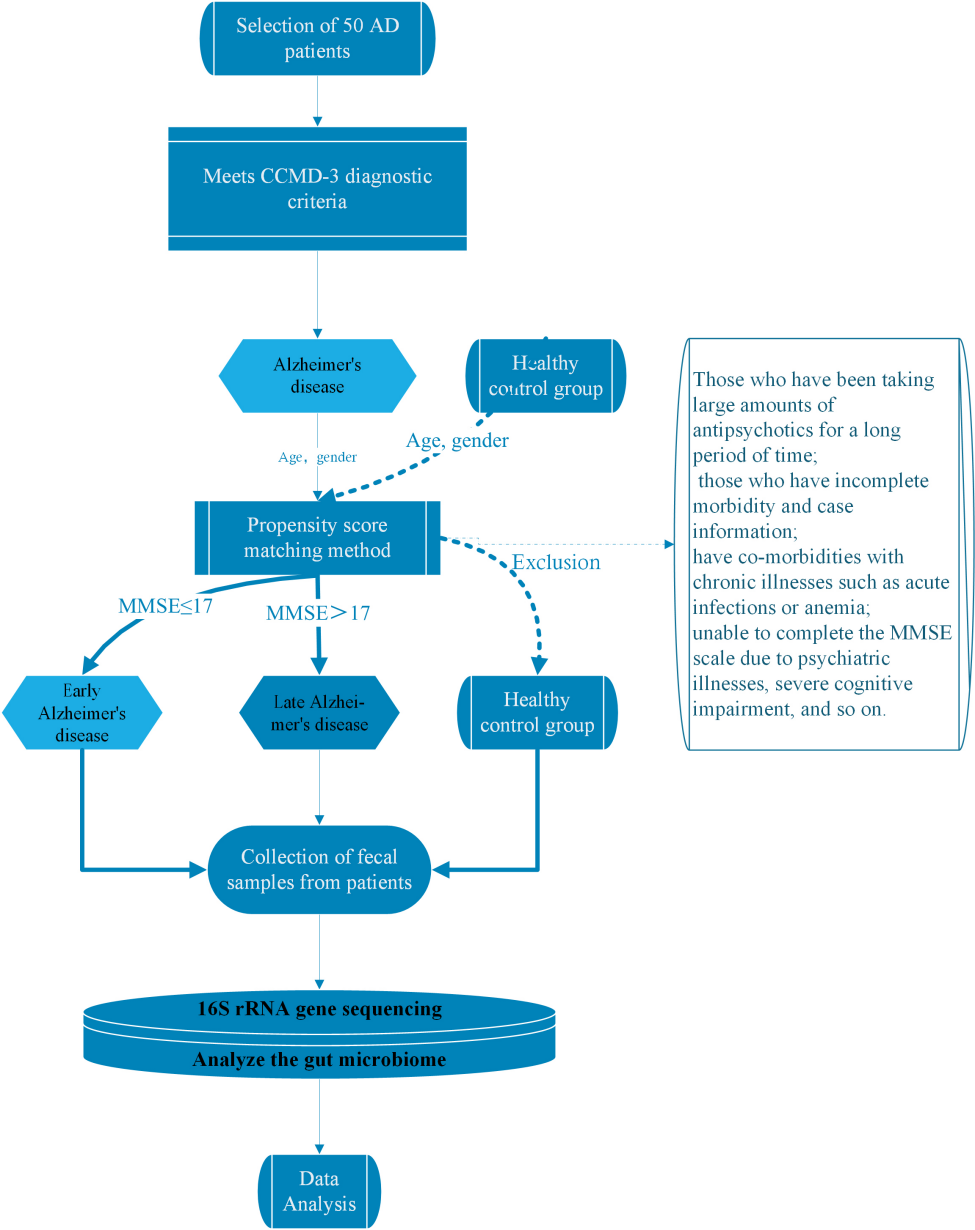
**Technology roadmap of this study**. Note: MMSE, Mini-Mental State Examination; CCMD-3, Chinese classification of mental disorders 3; AD, Alzheimer’s Disease.

Inclusion criteria for AD patients were as follows: Patients meeting the diagnostic criteria for AD according to the China Classification and Diagnostic Criteria for Mental Disorders (3rd Edition), individuals with a Mini-Mental State Examination (MMSE) score of 17 or lower were classified as having EAD [23], while those with higher scores were categorized as having Late Alzheimer’s Disease (LAD). This classification divides AD patients into EAD and LAD stages based on their disease progression. However, the healthy control group was selected based on the following criteria: MMSE score between 27 and 30, no history of cerebrovascular disease, and normal cognitive function. Moreover, the exclusion criteria included the use of antibiotics, immunosuppressants, proton pump inhibitors, non-steroidal anti-inflammatory drugs, anticonvulsants, antidepressants, and chemotherapeutic drugs (within 7 days before sample collection), consumption of high-fat and high-sugar diet, long-term heavy use of antipsychotic drugs, incomplete data, complication with chronic diseases (acute infection or anemia), and inability to complete the MMSE scale assessment due to mental illness or severe cognitive impairment. The study subject collection and enrollment process is shown in Fig. 1. Additionally, this study underwent a medical ethical review.

### Samples Collection

Before sampling, all participants were instructed to empty their bladders. A fresh fecal sample of about the size of a soybean was collected in a properly labeled sterile fecal sampler. Subsequently, the laboratory personnel dipped the sampling stick into the fecal sample and transferred it to a collection tube. After this, preservative fluid was blended with the fecal sample in the collection tube to form a homogenous suspension, which was then stored at –80 °C.

### DNA Extraction and Polymerase Chain Reaction Sequencing

The stool samples underwent four main steps: DNA extraction, PCR amplification, DNA sequencing, and data screening. Initially, DNA was extracted using the E.Z.N.A.® Soil Kit (Catalog No. D5625, manufactured by Omega Bio-tek, Norcross, GA, USA). The concentration and purity of DNA were assessed using a NanoDrop 2000 instrument, and the quality of DNA extraction was confirmed via 1% agarose gel electrophoresis. After this, the V3-V4 variable regions were amplified using primers 338F (5^′^-ACTCCTACGGGAGGCAGCAG-3^′^) and 806R (5^′^-GGACTACHVGGGTWTCTAAT-3^′^) [24]. After amplification, DNA sequencing was performed on the Illumina MiSeq PE 300 platform in the USA. Data sequencing included a quality control step to remove low-quality and duplicate sequences, and high-quality sequences were assembled into complete sequences. PCR conditions involved: initial denaturation at 95 °C for 30 seconds; denaturation at 95 °C for 15 seconds; annealing: reducing temperature to 55 °C for 30 seconds; extension at 72 °C for 45 seconds; cycle number: repeating steps 2–4 for a total of 35 cycles. A final extension of the DNA product was performed at 72 °C for 10 minutes to ensure complete synthesis of the target region, keeping the reaction at 4 °C until the next step.

### Data Processing

Sequencing data were converted to barcode information and then processed using the Quantitative Insights Into Microbial Ecology 2 (QIIME2) demux plugin (version 2021.2, QIIME2 development team, Flagstaff, AZ, USA) to demultiplex the feature sequences of the samples. Post-demultiplexing, the QIIME2 dada2 plugin was used for sequence quality control and chimera removal, strictly adhering to the quality control parameters of the QIIME2 platform. The QIIME2 feature-classifier plugin matches the representative sequences of sOTUs (sub-operational taxonomic units) against the pre-trained GREENGENES database (version 13-8, University of California, San Diego, CA, USA) with 99% similarity to obtain taxonomic classification. Contaminating mitochondrial and chloroplast sequences were eliminated using the QIIME2 feature-table plugin. Subsequently, the QIIME2 core-diversity plugin was used to compute diversity metrics, and the emperor plugin was employed to visualize the diversity. Differences in microbial abundance across groups and samples were assessed using various methods such as Analysis of Composition of Microbiomes (ANCOM), Analysis of Variance (ANOVA), Kruskal-Wallis test, Linear Discriminant Analysis Effect Size (LEfSe), and differential gene expression analysis utilizing the negative binomial distribution with DESeq2.

### Observation Indicators

General data: The medical staff of the hospital recorded general data of both patients and control groups, including gender, age, education level and Body Mass Index (BMI). The propensity score matching method was utilized to match AD with a control group at a 1:1 ratio.

Distribution of intestinal flora: A Venn diagram was created based on data sequencing to compare common species among different groups. Flora α diversity indicators were analyzed, such as Faith’s phylogenetic diversity (faith-pd) index, Chao1 richness estimator, Shannon diversity index (Shannon), and OTU index (Observed-otus).

Analysis of intestinal flora structure: Differences in intestinal flora across each group were assessed at genus, species, and phyla levels.

Correlation analysis between intestinal microflora and AD stage: Heat maps were used to analyze the correlation between different AD stages and intestinal microflora.

### Statistical Methods

Statistical analyses were performed using Origin 2021 software (Northampton, MA, USA). The normality of the data was tested using the Shapiro-Wilk test. Normally distributed quantitative data were described using the mean and standard deviation ($\bar{x}$ ± *s*), while non-normally distributed quantitative data were expressed as median (P25, P75). Comparisons between groups were performed using ANOVA or independent sample *t*-tests, with pairwise comparisons conducted using the Student-Newman-Keuls q (SNK-q) test. The χ^2^ test was used for intergroup comparisons of all categorical data.

Microbial community analysis employed the LEfSe test to differentiate relative abundances between groups, and difference was considered statistically significant when the logarithmic score of the linear discriminant analysis (LDA) log 10 >2, with *p* < 0.05 [25]. Furthermore, various alpha diversity indices were compared using the Wilcox analysis method, with an alpha correction level set at 0.05. Receiver operating characteristic (ROC) curves were utilized to identify biomarkers with significant differences for assessing the diagnostic efficacy of EAD. Beta diversity analysis was performed using the “fast.prcomp” function of the “gmodels” package (v.2.18.1) [26] in R software (R Foundation for Statistical Computing, Vienna, Austria) (v.4.2.2), and the results were visualized using the “ggpubr” package (v0.4.0) [27], a comprehensive web service for biomedical data analysis and visualization. Variables in the ROC curve analysis post-multivariate regression with results showing *p* < 0.05 and featuring an Area Under the Curve (AUC) >0.5, *p* < 0.05, and sensitivity generally >0.6 were considered diagnostically significant for EAD. We acknowledged the Hiplot Pro platform developed by Tengyun Biotech Co., Ltd., Shanghai, China (https://hiplot.com.cn/).

## Results

### General Data

The healthy control group consisted of 17 males and 33 females, aged between 54 and 91 years, with an average age of 75.44 ± 7.96 years and a body mass index (BMI) ranging from 16.06 to 30.71 kg/m^2^, averaging 21.84 ± 2.91 kg/m^2^. Regarding educational levels, 26 individuals had education up to middle school or lower, while 24 had attained high school or higher. The Alzheimer’s Disease (AD) patient group included 50 individuals, comprising 19 males and 31 females, aged between 59 and 92 years, with an average age of 74.80 ± 8.36 years and a BMI ranging from 16.93 to 27.78 kg/m^2^, averaging 22.44 ± 2.32 kg/m^2^. Educational levels were middle school or lower for 27 patients and high school or above for 23. There were no statistically significant differences in age, gender, BMI, or educational levels between the AD patients and the healthy controls (*p* > 0.05).

Among the AD patients, 14 were diagnosed with Early Alzheimer’s Disease (EAD) and 36 with Late Alzheimer’s Disease (LAD). In the EAD subgroup, there were 5 males and 9 females, aged between 60 and 85 years, with an average age of 75.07 ± 6.22 years and a BMI of 22.18 ± 1.99 kg/m^2^. Educational levels were middle school or lower for 9 individuals and high school or above for 5. The LAD subgroup consisted of 14 males and 22 females, aged between 60 and 85 years, with an average age of 74.69 ± 9.13 years and a BMI of 22.54 ± 2.45 kg/m^2^. Educational levels were middle school or lower for 18 individuals and high school or above for 18. There were no significant statistical differences in gender, age, or other general data between the EAD and LAD patients (*p* > 0.05). Baseline characteristics of the study subjects are given in Table 1.

**Table 1.** **Comparison of general information among the three experimental groups**.

Group (*n*)	Sex		Age (years)	Body mass index (kg/m^2^)	Educational qualifications (example)	
	Male	Female			Junior high school and below	High school and above
Healthy (50)	17	33	75.44 ± 7.96	21.84 ± 2.91	26	24
AD (50)	19	31	74.80 ± 8.36	22.44 ± 2.32	27	23
EAD (14)	5	9	75.07 ± 6.22	22.18 ± 1.99	9	5
LAD (36)	14	22	74.69 ± 9.13	22.54 ± 2.45	18	18
χ^2^_1_*/t*_1_	0.174		0.392	1.140	0.040	
*p*_1_	0.677		0.696	0.257	0.841	
χ^2^_2_/*F*	0.218		0.087	0.745	0.866	
*p*_2_	0.897		0.917	0.478	0.649	

Note: “χ^2^_1_” refers to the chi-square test comparing the Healthy group and the Alzheimer’s Disease (AD) group. “*t*_1_” denotes the *t*-test conducted between the Healthy group and the AD group. “*p*_1_” represents the *p*-value for the comparison between the Healthy and AD groups. “χ^2^_2_” indicates the chi-square test comparing the Healthy, Late Alzheimer’s Disease (LAD), and Early Alzheimer’s Disease (EAD) groups. “*F*” represents the Analysis of Variance (ANOVA) results when comparing the Healthy, LAD, and EAD groups. “*p*_2_” is the *p*-value for comparing the Healthy, LAD, and EAD groups. Here, ‘Healthy’ pertains to the group without Alzheimer’s Disease, ‘EAD’ to the group with Early Alzheimer’s Disease, and ‘LAD’ to the group with Late Alzheimer’s Disease.

### Analysis of Beta Diversity among Intestinal Flora

**Fig. 2.**
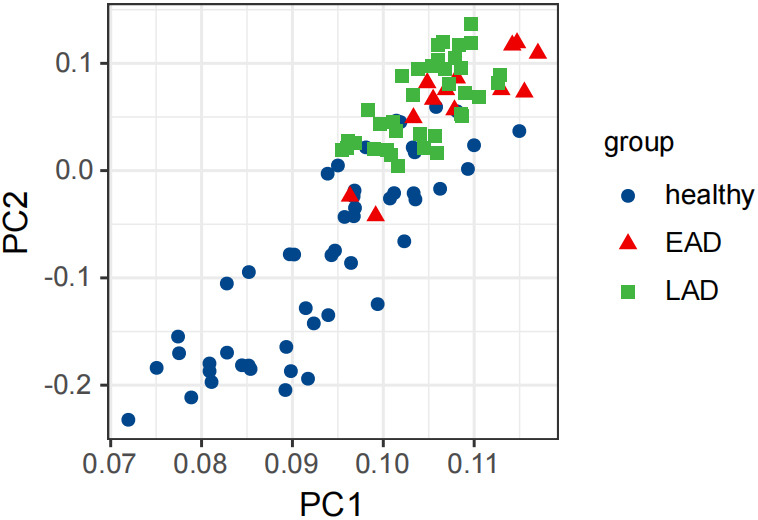
**Principal Components Analysis (PCA) differentiation scenario**. Note: The X-axis: PC1, indicates the first principal component; the Y-axis: PC2, represents the second principal component; “healthy”, “EAD”, and “LAD” represent the healthy control group, Early Alzheimer’s Disease, and Late Alzheimer’s Disease, respectively.

The variation explained by PC1 was 12.12% and by PC2 was 7.94%. PCA analysis effectively distinguished between the microbial structures of the EAD group and those of ‘healthy’ and ‘LAD’ groups, as shown in Fig. 2.

### Sequencing Data and Analysis of Common Strains

The 100 fecal samples from the EAD, LAD, and healthy control groups were analyzed using 16S rRNA sequencing, yielding 2,458,406 usable sequences with an average length of 412 bp. After clustering and denoising processes, 2523 operational taxonomic units (OTUs) were identified. These OTUs were classified into 11 phyla, 20 classes, 32 orders, 65 families, and 132 genera.

**Fig. 3.**
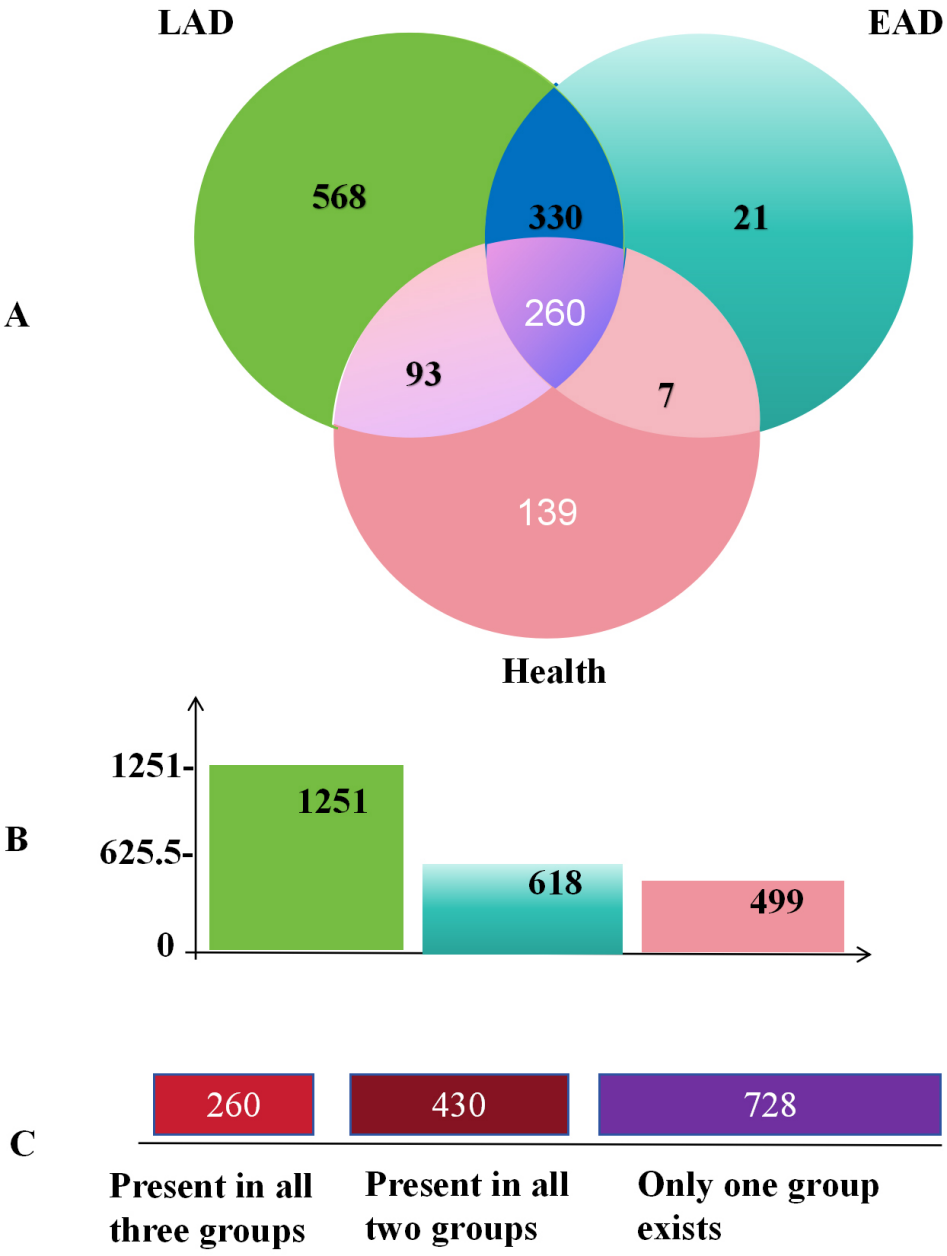
**Venn diagrams of common and endemic species among the three experimental groups**. Note: (A) The Venn diagram of shared and unique species among the three groups. (B) The number of bacterial species in each group. (C) The 1418 effective operational taxonomic units (OTUs).

Sequences of OTUs with an abundance >1‰ were selected and aligned to the sequence similarities in the GREENGE-NES Database, version 13-8 ribosomal RNA database for species annotation information. This step primarily aimed to obtain the absolute abundance and annotation information of OTU and to count the ratio of the number of sequences at the species, genus, family, and phylum levels to the total number of sequences for each sample. As illustrated in Fig. 3, a total of 1418 effective OTUs were selected, with 360 bacterial species shared between AD patients and healthy controls, and 590 shared between EAD and LAD patients. Furthermore, EAD patients had 21 unique bacterial species.

### Analysis of Microbial Diversity

**Fig. 4.**
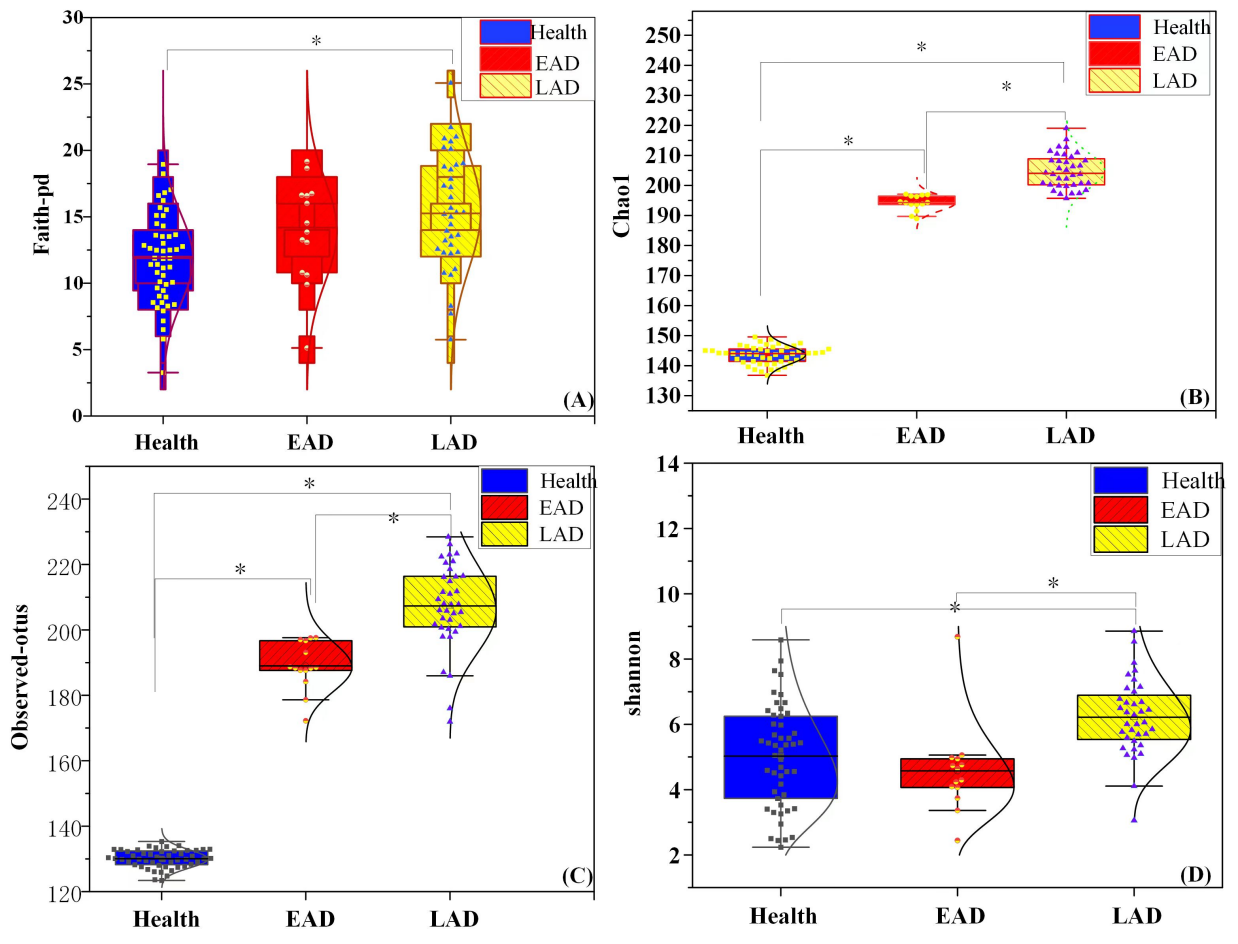
**Comparison of α diversity indicators among the three experimental groups**. Note: (A) Box plot of three groups of faith-pd index. (B) Box plot of three groups of chao1 index. (C) Box plot of observed-otus index. (D) Box plot of three groups of Shannon index. ^*^p < 0.05.

Patients in the EAD and LAD groups demonstrated significantly higher values compared to the control group in metrics such as Faith’s phylogenetic diversity, Chao1 index, Observed OTUs index, and Shannon index, with significant statistical differences (F = 2486.926, 925.016, 10.248; *p* < 0.001 for all indices). However, only the Chao1 and Observed-OTUs indexes showed significant differences in pairwise comparisons among the three groups (*p* < 0.05, Fig. 4).

### Analysis of Gut Microbiome Composition at the Genus Level

**Fig. 5.**
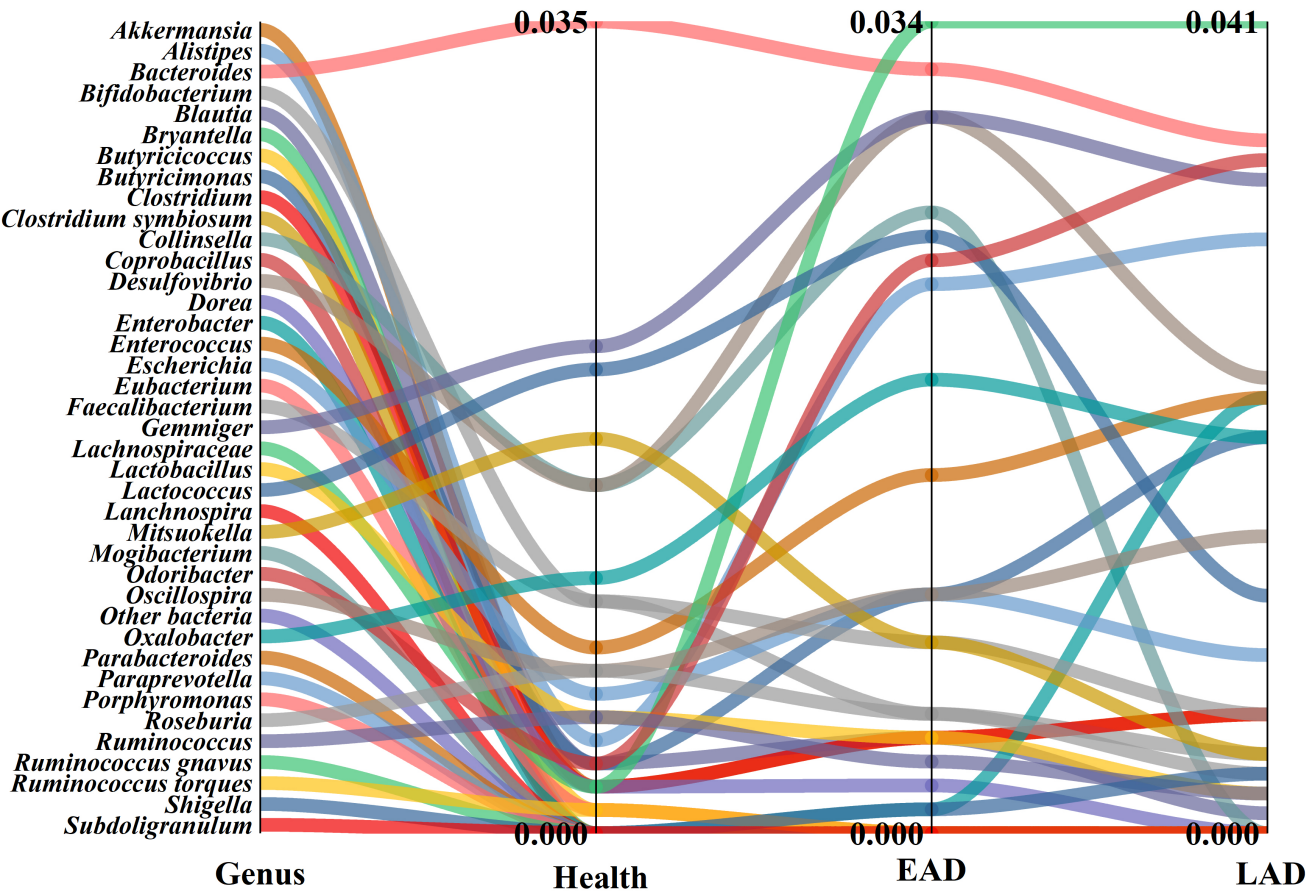
**Comparison of bacterial abundance among the three experimental groups at the genus level**. Note: The left side of the figure indicates the genus, other bacteria represent the bacterial abundance of other genera. Health, EAD, and LAD represent the changes of the three groups of bacteria, respectively. The numbers in the figure represent the minimum and maximum abundance of bacterial species.

Among the 132 genera analyzed at the genus level, significant differences between AD patients and the healthy control group were primarily observed in 39 genera. The predominant genera in the healthy control group included *Gemmiger*, *Bacteroides*, and *Mitsuokella*. In the EAD group, there was a substantial increase in the relative abundance of genera such as *Bryantella*, *Gemmiger*, *Desulfovibrio*, *Collinsella*, *Odoribacter*, and *Enterococcus *compared to the healthy controls. Furthermore, the relative abundances of *Lactococcus*, *Gemmiger*, and *Desulfovibrio* were higher in the EAD group compared to the LAD group. These differences were assessed using the LEfSe test (all *p* < 0.05, Fig. 5).

### Structural Analysis of Intestinal Flora at the Phylum and Order Level

As shown in Table 2, the main differences among the EAD, LAD, and healthy control groups were observed in the phyla *Actinobacteria*, *Bacteroidetes*, *Firmicutes*, *Proteobacteria*, and *Verrucomicrobia*, as well as in the orders *Clostridia*, *Enterobacteriales*, *Desulfovibrionia*, *Verrucomicrobiales* and* Lactobacillales.* These differences were statistically significant (*p* < 0.01).

**Table 2.** **Comparison of dominant bacterial abundance among the three experimental groups**.

Phylum, order	Relative abundance (%)			log 10 LDA	*p*
	Healthy control group	EAD	LAD		
*Actinobacteria*	0.03 (0.01, 0.74)	0.34 (0.23, 0.67)	0.44 (0.31, 0.64)	4.98	0.003
*Firmicutes*	0.64 (0.52, 0.75)	0.73 (0.70, 0.75)	0.76 (0.70, 0.74)	4.72	0.004
*Proteobacteria*	0.12 (0.10, 0.15)	0.06 (0.05, 0.07)	0.04 (0.03, 0.05)	5.32	0.002
*Bacteroidetes*	0.06 (0.05, 0.07)	0.08 (0.07, 0.09)	0.09 (0.08, 0.10)	3.92	0.006
*Verrucomicrobia*	0.03 (0.02, 0.04)	0.01 (0.01, 0.02)	0.03 (0.02, 0.03)	4.12	0.005
*Clostridiales*	0.65 (0.60, 0.71)	0.58 (0.52, 0.64)	0.52 (0.50, 0.64)	3.58	0.008
*Enterobacteriales*	0.11 (0.10, 0.14)	0.08 (0.07, 0.09)	0.04 (0.02, 0.05)	7.24	0.001
*Desulfovibrioales*	0.03 (0.01, 0.03)	0.01 (0.01, 0.02)	0.05 (0.04, 0.06)	5.82	0.002
*Verrucomicrobiales*	0.03 (0.02, 0.04)	0.01 (0.01, 0.02)	0.02 (0.02, 0.03)	3.98	0.006
*Lactobacillales*	0.01 (0.01, 0.02)	0.06 (0.05, 0.07)	0.08 (0.07, 0.09)	8.14	0.000

Note: EAD, Early Alzheimer’s Disease; LAD, Late Alzheimer’s Disease; log 10 LDA, log-transformed Linear Discriminant Analysis.

### Discriminatory Analysis of Intestinal Microflora and EAD Staging

**Fig. 6.**
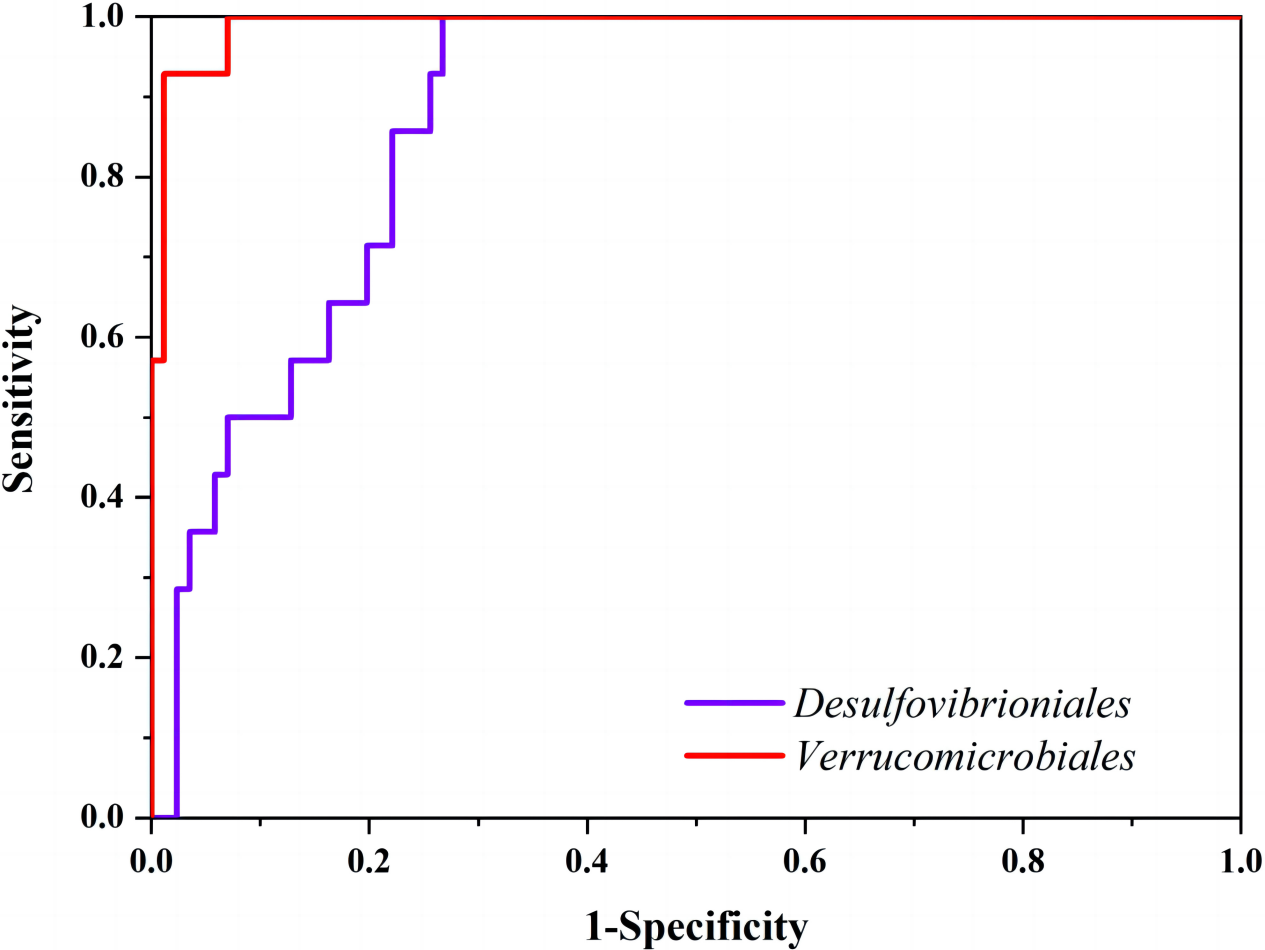
**Comparison of receiver operating characteristic curves for EAD using main difference indicators of intestinal flora**. Note: The X-axis represents 1-Specificity, which is the proportion of test results that incorrectly identify healthy individuals as patients. The Y-axis represents sensitivity, which is the proportion of actual positive samples that are correctly predicted as positive. The curve shows the relationship between sensitivity and specificity at different thresholds. The closer the curve is to the upper left corner, the better the predictive performance of the model.

Multivariate linear regression analysis revealed that the faith-pd index, *Enterobacteriales*, *Desulfovibrionia*, *Verrucomicrobiales*, and *Lactobacillales* demonstrated a significant impact on EAD and LAD (F = 690.004, *p* = 0.000). These results are presented in Table 3. Using EAD as the implicit variable, with EAD as positive and Healthy and LAD as negative, the discriminating variables included the Faith-PD index, *Enterobacteriales*, *Desulfovibrionaceae*, *Verrucomicrobiales*, and *Lactobacillales*. Receiver operating characteristic (ROC) curves illustrating these findings are displayed in Fig. 5. Indicators with *p* < 0.05 were used to identify the intestinal flora indicators of EAD patients, such as the observed-otus index and the relative abundance of *Enterobacteriale*, *Desulfovibrionia*, *Verrucomicrobiales*, *Lactobacillales* (Table 4). The ROC curve results revealed that an index only has a diagnostic value when AUC >0.50, *p* < 0.05, and sensitivity >0.6. ROC curves (Fig. 6) indicated that only *Desulfovibrionales* and *Verrucomicrobiales* met the criteria as mentioned above and were found suitable for clinical diagnostics.

**Table 3.** **Multivariate linear regression analysis of AD**.

Model	B	SE	Standardized coefficient beta	*t*	*p*
Constant	0.266	0.517		0.514	0.609
Faith_pd	–0.008	0.003	–0.034	–2.689	0.009
Chao1	0.004	0.002	0.118	1.615	0.110
Observed_otus	0.001	0.001	0.051	1.034	0.304
Shannon	0.006	0.007	0.010	0.884	0.379
*Desulfovibrio*	–0.907	0.83	–0.063	–1.093	0.278
*Gemmiger*	–1.246	0.861	–0.121	–1.447	0.152
*Lactococcus*	0.008	0.741	0.001	0.011	0.991
*Actinobacteria*	0.050	0.053	0.010	0.942	0.349
*Bacteroidetes*	–0.216	0.226	–0.016	–0.958	0.341
*Firmicutes*	–0.004	0.911	0.000	–0.005	0.996
*Proteobacteria*	0.504	1.632	0.008	0.308	0.759
*Verrucomicrobia*	3.551	2.014	0.027	1.763	0.082
*Clostridiales*	–0.286	0.253	–0.019	–1.128	0.263
*Enterobacteriales*	–6.492	0.952	–0.318	–6.817	0.000
*Desulfovibrionia*	8.23	1.434	0.135	5.739	0.000
*Verrucomicrobiales*	5.407	1.874	0.041	2.886	0.005
*Lactobacillales*	8.200	1.974	0.266	4.153	0.000

SE, standard error.

**Table 4.** **ROC curves of the main indicators of variation in intestinal flora for Early Alzheimer’s Disease (EAD)**.

Indicators of difference	AUC	Critical value	Sensitivity	Specificity	95% CI	*p*-value
*Desulfovibrioales*	0.854	0.019	92.86	73.26	0.722∼0.986	<0.001
*Verrucomicrobiales*	0.966	0.020	92.86	100.00	0.930∼1.001	<0.001

Note: ROC, receiver operating characteristic; AUC, Area Under the Curve.

## Discussion

In this study, we used 16S rRNA sequencing technology to systematically and comprehensively analyze the intestinal flora composition of AD patients and controls at different stages. This study aimed to explore the significance of this method for early diagnosis of AD. The findings demonstrated that EAD patients exhibited significantly increased gut microbial abundance and diversity compared to healthy controls. Furthermore, substantial differences were found in the relative abundance of specific genera, suggesting possible biological markers for AD. The 16S rRNA technique is simple, cost-effective, and effectively detects changes in gut microbial diversity, especially the *Verrucomicrobiales* order, underscoring its potential for clinical translational applications [28]. This study also identified potential predictive factors for EAD, such as the observed-otus index and changes in the actinobacteria phylum, which showed some abilities to distinguish EAD. AD is believed to be caused by the accumulation of extracellular β-amyloid and intracellular tau. Several studies have shown that treatment with β-amyloid binding antibodies reduces brain amyloid levels and decreases cognitive function [29, 30]. Furthermore, AD is accompanied by intestinal cholinergic dysfunction and gastrointestinal issues [31]. Currently, AD diagnosis relies on neuropsychological assessment, but the sensitivity and specificity are inadequate, and there is a lack of biological markers for early diagnosis. It was observed that an isolated central AD-like neuropathology results in disrupted homeostatic control of Gastrointestinal (GI) tract outflow via the BG axis, potentially increasing susceptibility in the Streptozotocin-Intracerebroventricular (STZ-ICV) rat model of AD to systemic inflammation induced by gastrointestinal and intraluminal toxin, microbial, and drug [32]. Elucidating the intestinal flora status of patients is helpful in determining their cognitive function and the degree of intestinal dysfunction.

Compared to previous studies, this study observed EAD, healthy control and LAD based on 16s rRNA detection technology to determine the intestinal microbial dysbiosis of AD patients. A study previously reported that surgical procedures exacerbate existing microbial dysbiosis and intestinal barrier dysfunction in patients with pro-Alzheimer’s disease phenotype, leading to further cognitive deterioration [33]. Furthermore, a study using quantitative Polymerase Chain Reaction (qPCR) of bacterial 16S rDNA genes has examined the composition of oral bacterial flora, indicating a significant correlation between changes in oral microbial flora, increases in inflammatory cytokines and AD [34]. Furthermore, it has been shown that AD-related alterations are not confined to the brain but extend throughout the gastrointestinal tract, including immune and neuronal changes [35]. In recent years, researchers have made considerable progress in delaying the onset of AD through interventions targeting gur microbiota dysbiosis and have indicated SLAB51’s ability to exert neuroprotective and anti-inflammatory effects in AD models, demonstrating a novel mechanism [36]. In AD research, alterations in the gut microbiome and an increase in intestinal inflammation may result from pathways other than SIBO and are more complex than bacterial overgrowth in the small intestine [37]. Interestingly, no correlation has been observed previously between SIBO and stool calcin levels in AD patients [38]. Moreover, research has revealed that Aβ or Tau fibrils injected into the colon or brain lysates from AD patients can diffuse from the intestine to the brain via the vagus nerve, triggering AD pathology and cognitive dysfunction [39]. These studies provide strong evidence of disrupted gut microbiota in patients with AD, highlighting the need for specific investigation into EAD.

The sample size and grouping criteria selected in this research were similar to ours, and the identified predictive factors differed: they found significant changes in the genera Acetivibrio and Bifidobacterium, along with a decrease in gut diversity [40]. The study primarily focused on patients with Parkinson’s disease. In investigations involving the elderly population, researchers have demonstrated changes in the gut microbiome associated with aging, such as a reduction in the proportion of *Firmicutes* and *Bifidobacteria* counts [41]. Furthermore, animal study have revealed that aging mice with cognitive impairments exhibit abnormal fecal microbiota, characterized by higher microbial α-diversity, increased *Firmicutes/Bacteroidetes*ratios, and elevated levels of pathogenic bacteria [42]. Various gut microbes, such as *Actinomycetes*, *Bacteroides*, *Escherichia coli*, *Firmicutes*, *Proteobacteria*, *Tenericutes*, and *Verrucomicrobia*, play crucial roles in the pathogenesis of AD [43]. Some researchers have indicated the prevalence of Bacillus and acidophilic bacteria in AD cases [44]. However, comprehensive conclusions regarding assessments across various stages of AD are yet to be explored. Our results indicated a significant increase in the relative abundance and α-diversity of genera such as *Bryantella* in both early and late stages of AD. The differences observed are likely due to variations in sample demographics and timing. Additionally, assessing microbial functional aspects could further elucidate the physiological mechanisms underlying gut microbiota dysregulation.

For instance, a study has investigated the effects of 8 weeks of moderate-intensity aerobic exercise, consumption of chlorogenic acid, and their combination on Aβ deposition, inflammatory markers, oxidative stress biomarkers, neuronal damage, and cognitive abilities in AD mice model, revealing that lifestyle changes can aid in improving quality of life in AD-related conditions [45]. Furthermore, recent research using transcriptome sequencing has confirmed that inhibiting the Extracellular Signal-Regulated Kinase (ERK)/Freiberg-Berk-Jenson Osteosarcoma Oncogene (FOS) axis to alleviate inflammation can reverse the pathological symptoms of AD [46]. From a pathophysiological perspective, cognitive impairments and biomarkers in AD patients vary across various stages [47]. Therefore, it is reasonable to believe that utilizing 16S rRNA to examine gut microbiota in patients can improve the current challenges in early recognition of EAD, underscoring the significance of this research.

Based on the above findings, this study identifies certain limitations that offer opportunities for reference and improvement to future studies. Given the highly heterogeneous nature of AD, larger sample sizes allow a more comprehensive assessment of differences among subpopulations. Additionally, the analysis of 16S rRNA in this study was limited to structural levels, implying that future studies could integrate genomic and transcriptomic data to unravel microbial differences at functional and mechanistic levels. Simultaneously, there is a need to improve the clinical database to explore the correlation between microbial markers and disease progression and disintegration. Future research could also involve animal experiments to validate the role of specific bacterial species on AD through comparative group studies. Overall, this study establishes a foundation for diagnosing EAD based on intestinal microbiota. However, the sample size and follow-up time should be increased. It is believed that comprehensive research will expand our biological understanding and improve AD diagnosis. These limitations could restrict the universality and statistical robustness of the findings. Moreover, the on-random sample selection complicates the ability to fully represent the entire population, thus affecting the extrapolation of results. The relatively short sample collection period hinders the evaluation of long-term dysbacteriosis in the intestinal flora. In the future, using high throughput approaches and larger sample sizes, it is anticipated that a more comprehensive and systematic understanding will be obtained. This study is expected to substantially promote the practice of early diagnosis and precise treatment of AD.

## Conclusion

By systematically analyzing and comparing the fecal microbial compositions of early and late-stage AD patients with healthy controls, this research reveals that 16S rRNA technology can effectively differentiate EAD patients, potentially offering novel biological insights. Furthermore, this study successfully identifies predictive factors within the gut microbiota, such as the observed-otus index and changes in *Actinobacteria* and *Verrucomicrobiales*, suggesting their potential application in diagnosing EAD. These findings provide a reference for subsequent large-scale studies, fostering precise diagnosis of EAD and contributing to the development of novel therapeutic strategies targeting the microbiota, thus supporting early identification and drug development for EAD.

## Availability of Data and Materials

The data used and/or analyzed during the current study are available from the corresponding author.
